# The Obstacles to Current Extracellular Vesicle-Mediated Drug Delivery Research

**DOI:** 10.15436/2377-1313.17.1331

**Published:** 2017-10-27

**Authors:** Heedoo Lee, Duo Zhang, Ashish Rai, Yang Jin

**Affiliations:** 1 Division of Pulmonary and Critical Care Medicine, Department of Medicine, Boston University, Boston, MA, USA; 2 Internal Medicine, Salem Hospital, North Shore Medical Center, 81 Highland Ave, Salem, MA

**Keywords:** Extracellular vesicles, Microrna, Drug delivery

## Abstract

Extracellular Vesicles (EVs) are nanometer-sized cell-derived membrane vesicles that are released by donor cells and play an important role in intercellular communication. In this short communication, we discuss the obstacles currently faced in EV-mediated drug delivery research. The commonly used vehicle for drug delivery in prevalent practice are liposome’s which are synthetic vesicles, these vesicles commonly interact with serum proteins, macrophages and other innate immune response molecules and may be destroyed before they can deliver the drug. EVs however have the same membrane compositions and similar cell surface markers as the cells from which they are derived which thus prevents interactions or provocations of an immune response. In addition, EVs have been used to deliver molecules across tight cellular junctions such as the blood brain barrier. This has led to an interest in using EVs as a novel method for drug delivery. We hereby discuss the potential pitfalls and difficulties that need to be addressed before EVs can be used as drug delivery vehicles in pharmacological research.

## Introduction

Extracellular Vesicles (EVs) are nanometer-sized cell-derived membrane vesicles, released by many cell types. They primarily function as intercellular communication tools and ferry proteins, lipids and nucleic acids. EVs are generated through a dynamic process and they are classified into three main groups on the basis of their intracellular origin as, Apoptotic Bodies (ABs), Micro Vesicles (MVs) or exosomes (Exos) ^[1]^ (Schema 1). The three classes of EVs differ mainly in size and the mechanism of biogenesis. Exosomes are thought to be the smallest vesicle type, with sizes ranging from 40 to 100 nm. MVs are larger, ranging from 50 nm to 1 μm and can form through direct budding from the plasma membrane. ABs are one of the largest EVs, about 1 – 5 μm and these are released when cells undergo apoptosis^[1]^. Since ABs are essentially a by-product of cellular death, they are considered less useful for drug delivery.

The prevalent vehicle for drug delivery, liposomes, can be loaded with small molecules or nucleic acids and have been in use for decades. EVs and liposomes are similar in constitution and are made up of a lipid bilayer with an aqueous inner layer. On average liposomes have a size of ~30 nm, which is similar to that of Exos. The liposomal surface is usually modified to reduce interactions with serum proteins, unlike EVs which already carry surface proteins that avoid any such interaction^[2]^. The surface of EVs is thought to be adapted to avoid detection by the immune system and to promote uptake by specific cell types. Given this advantage, EVs are more efficient than the synthetic liposome’s in drug delivery to a cell. They can deliver molecules even through hard-to-cross barriers like the blood-brain barrier as they carry the same membrane compositions as that of the blood vessels^[3]^. Previous reports show that EVs can be loaded with potent pharmacological agents such as curcumin, cucurbitacin-I, doxorubicin and paclitaxel^[4]^. The initial cargos of EVs comprised of small and long, coding and non-coding RNAs (mRNA, miRNA, lncRNA), lipids and proteins. These RNA and proteins that are transported by EVs could be devised as potential drugs for therapeutic purposes.

One of the preliminary challenges in working with EVs is the difficulty in identifying and isolating them accurately primarily because of the overlap in their sizes^[5]^. Protein biomarkers such as CD9, CD63, CD81 which are associated with the Exos, and CD63, CD9 found on the MVs and Abs (6) are commonly used to distinguish between the EVs. Other classic markers like flotillin-1, heat-shock protein HSC70, and MHC class I and II that are currently used as markers, are expressed at the same level in all the three kinds of EVs^[6]^ creating a limited way to differentiate between EVs.

### EVs in RNA delivery

Micro RNAs (miRNAs) are the most widely studied family of non coding RNA with 17 – 22 nucleotides (nt). They regulate post-transcriptional gene expression and have critical roles in basic biological processes. Dysregulation of miRNAs can lead to disease states such as cancer. The chemically modified miRNAs are more stable as compared to other RNAs. Additionally, they are easier to synthesize and manipulate as they are very short in length. miRNAs can bind with mRNA to target mRNA destabilization, translational repression and activation of gene expression, and one miRNA may regulate multiple genes as its targets^[7]^. This makes miRNAs a potential therapeutic agent and EVs would be the ideal vehicle for delivering them to the cell. However, there are several problems that need to be addressed before this can become a reality.

### Potential problems to deliver miRNA using EVs

1. Amount of specific miRNAs in one exosome: less than one copy per exosome^[8]^. At least 100 exosomes would be needed to find one copy of a given miRNA if we consider a homogenous distribution^[8]^. This stoichiometry of miRNAs and exosomes suggests that most individual exosomes in standard preparations do not carry biologically significant numbers of miRNAs and therefore, are individually unlikely to be functional as vehicles for miRNA-based communication.
2. No efficient way to determine the delivery efficacy and efficiency of Exos: Exos are generally labelled with fluorescent dye and the dye on the recipient cell is checked for delivery. Introduction of the miRNAs into EVs using lipofection or electroporation is common. Since the liposome with miRNAs can also be transfected into recipient cells and freely circulating miRNAs in the cell culture medium may also have functions, it is hard to accurately represent the efficiency of EVs mediated mechanism^[9]^. Similarly, efficacy is usually examined by recipient cells protein expression using western blot analysis, and this is also an indirect method of measuring the end result^[9]^.
3. The most important problem that persists is that of cell specific delivery. Similar to most drugs and drug vehicles, a majority of EVs are taken up by cells through nonspecific methods such as endocytosis, macropinocytosis and phagocytosis^[2]^. Since EVs are the cell’s communication tools, we believe that modified surface markers could make the EVs deliver to specific cells. For example, using CD47 markers can block the programmed cell removal signal to macrophages^[10]^.

### EVs in protein delivery

In addition to delivering small RNAs, EVs can be also used to deliver large molecules such as proteins. There are however only a limited number of studies that have looked at protein enriched EVs. One study showed that when exosomes enriched with the enzyme catalase was successfully transported across the blood–brain barrier it lead to improvement in Parkinson’s disease symptoms^[11]^. However, size continues to be a limitation, especially Exos which are only 100 nm in maximum dimensions and may be too small to carry significant amount of protein. The structure of protein is more complicated than miRNAs and plays a key role in its function. Proteins after being enriched in EVs may lose their secondary structures (helices, β-sheets and β-barrels) and may not be functionally viable anymore.

## Conclusion

In conclusion, drug delivery through extracellular vesicles is becoming more popular in the medical field. Even though they have several advantages compared to liposomes and other nanoparticles, such as lower toxicity and subdued immune response the road to proficient use of EVs for drug delivery is fraught with hurdles. For miRNA, these include inability to determine the exact copy number, the delivery efficacy or efficiency and inability to deliver EVs specifically to certain cell types, and for the EV-mediated proteins, it is the inability to maintain intact structure during transport and to hold large proteins without altering their secondary structure.

## Figures and Tables

**Schema 1:**
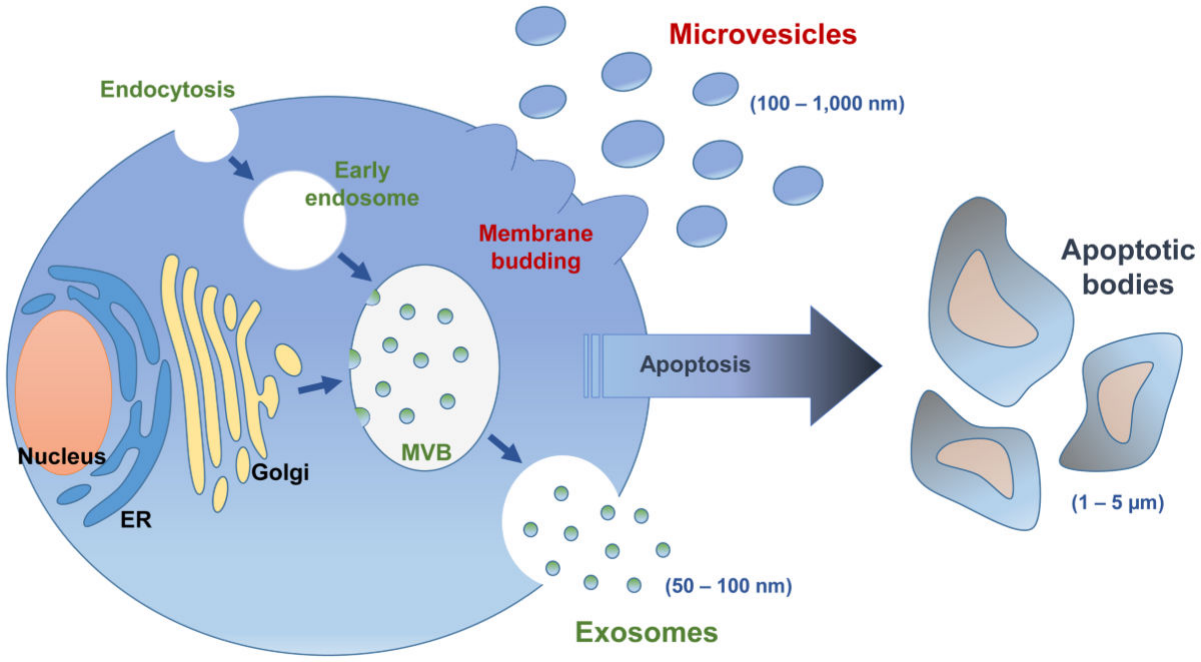


## References

[1] Yáñez-Mó M, Siljander PR, Andreu Z (2015). Biological properties of extracellular vesicles and their physiological functions. J of extracellular vesicles.

[2] Mulcahy LA, Pink RC, Carter DRF (2014). Routes and mechanisms of extracellular vesicle uptake. Journal of extracellular vesicles.

[3] Lydia Alvarez-Erviti, Seow Y, Betts C (2011). Delivery of siRNA to the mouse brain by systemic injection of targeted exosomes. Nature biotech.

[4] Kotmakćı M, Çetintaş VB (2015). Extracellular vesicles as natural nanosized delivery systems for small-molecule drugs and genetic material: steps towards the future nanomedicines. J Pharm Pharm Sci.

[5] Gould SJ, Raposo G (2013). As we wait: coping with an imperfect nomenclature for extracellular vesicles. J of extra cell vesicles.

[6] Kowal J, Arras G, Colombo M (2016). Proteomic comparison defines novel markers to characterize heterogeneous populations of extracellular vesicle subtypes. Proc Natl Acad Sci U S A.

[7] Huntzinger E, Izaurralde E (2011). Gene silencing by microRNAs: contributions of translational repression and mRNA decay. Nat Rev Gen.

[8] Chevillet JR, Kang Q, Ruf IK (2014). Quantitative and stoichiometric analysis of the microRNA content of exosomes. Proc Natl Acad Sci U S A.

[9] Ha D, Yang N, Nadithe V (2016). Exosomes as therapeutic drug carriers and delivery vehicles across biological membranes: current perspectives and future challenges. Acta Pharm Sin B.

[10] Feng M, Chen JY, Weissman-Tsukamoto R (2015). Macrophages eat cancer cells using their own calreticulin as a guide: roles of TLR and Btk. Proc Natl Acad Sci U S A.

[11] Matthew J Haney, Klyachko Natalia L, Zhao Y (2015). Exosomes as drug delivery vehicles for Parkinson’s disease therapy. J of Controlled Release.

